# Normalization using ploidy and genomic DNA copy number allows absolute quantification of transcripts, proteins and metabolites in cells

**DOI:** 10.1186/1746-4811-6-29

**Published:** 2010-12-29

**Authors:** Hiroshi Shimada, Takeshi Obayashi, Naoki Takahashi, Minami Matsui, Atsushi Sakamoto

**Affiliations:** 1 Department of Mathematical and Life Sciences, Graduate School of Science, Hiroshima University, 1-3-1, Kagamiyama, Higashi-Hiroshima, 739-8526, Japan; 2 Graduate School of Information Sciences, Tohoku University, 6-3-09, Aramaki-Aza-Aoba, Aoba-ku, Sendai 980-8579, Japan; 3 Plant Functional Genomics Research Group, Plant Science Center, RIKEN Yokohama Institute, 1-7-22 Suehiro-cho, Tsurumi-ku, Yokohama, 230-0045 Japan

## Abstract

**Background:**

Quantification of transcripts, proteins, or metabolites is straightforward when the factor used to normalize these values remains constant between samples. However, normalization factors often vary among samples and thus must be developed for each new analytical method.

**Results:**

We demonstrate quantification of transcript and protein levels in Arabidopsis based on genomic DNA copy number. We extracted total nucleic acid from 3-week-old rosette leaves of wild-type Arabidopsis and the pale-green/dwarf mutant, *abc4*, and quantified the number of transcripts by quantitative reverse-transcription PCR using genomic DNA copy number and ploidy (as determined by cytometry) for normalization. Our data indicated that normalization using genes commonly employed as references resulted in inaccuracies in transcript levels of the genes *RBC-L *and *RBC-S *(encoding the large and small subunits, respectively, of ribulose 1,5-bisphosphate carboxylase/oxygenase) in wild type and mutant. Normalization using genomic DNA copy number and ploidy, however, appropriately showed that the *RBC-L *and *RBC-S *transcript levels per cell in the mutant were significantly lower than that in wild type. Furthermore, quantification revealed that a cell of a 3-week-old wild-type Arabidopsis rosette leaf had an average of 7.5 × 10^3 ^transcripts of *RBC-L*, 9.9 × 10^3 ^transcripts of *RBC-S*, and 1.4 × 10^6 ^*18S rRNA*. We similarly analyzed the accumulation of RBC-L and LHCP (light-harvesting chlorophyll *a/b *protein) in wild type and mutant based on ploidy and genomic DNA copy number that was determined by direct quantitative PCR analysis of extracts using a DNA polymerase tolerant to a wide range of common PCR inhibitors. Furthermore, we estimated the number of RBC-L molecules (2.63 × 10^8^) and chlorophyll molecules (1.85 × 10^9^) in each cell in 3-week-old wild-type rosette leaves; these values had relatively low coefficients of variation, underscoring the reliability of our method.

**Conclusion:**

Genomic DNA copy number and ploidy are useful as general normalization factors, providing an easy method for determining the number of transcripts, proteins, and metabolites in a cell.

## Background

Cellular levels of transcripts, proteins, and metabolites are usually quantified relative to the value for a known, constitutively expressed cellular factor. Quantification of transcripts using northern hybridization is based on total amounts of RNA or mRNA. Quantification of transcripts using RT-PCR analysis, including real-time RT-PCR, is based on the expression level of a reference gene [1-4], and a DNA array detects relative levels of transcripts [5,6]. Protein levels are typically quantified by Coomassie Brilliant Blue (CBB) staining of samples subjected to SDS-PAGE, by two-dimensional difference gel electrophoresis for proteome analysis, by immunoblotting, or by enzyme-linked immunosorbent assay relative to the weight of total protein, fresh weight, dry weight or culture volume. Metabolites are often quantified based on the weight of total protein, fresh weight or dry weight. Such quantification methods are useful when the normalization factor does not vary among samples. Between tissues, however, the transcriptional activity may differ, and the ratio between mRNA and rRNA may vary widely depending on the cell population [7,8]. Because rRNA comprises a large proportion of total RNA in the cell, transcript quantification based on total amounts of RNA or mRNA in one cell type may not accurately reflect the transcript levels in other cell types. The precision of quantitative (q)RT-PCR depends on accurate transcript normalization using constitutively expressed genes. Statistical algorithms have been developed to help validate reference genes [3,4]; prior to analysis, however, it is difficult to know which reference gene is consistently expressed among the samples, such as when a novel mutant or treatment analysis is under consideration. Similarly, total protein, fresh weight, dry weight, or culture volume may vary between samples.

In *Arabidopsis thaliana*, *abc4 *is a mutant of the phylloquinone biosynthesis gene and exhibits the dwarf and pale-green phenotype [9]. The mutant has fewer chloroplasts than wild type, and the intercellular space is also larger [9]. Northern hybridization using total RNA revealed that the *RBC-L *(Rubisco large subunit) and *RBC*-*S *(Rubisco small subunit) transcript levels are significantly elevated in the *abc4 *mutant, whereas the *LHCP *(light-harvesting chlorophyll *a/b *protein) transcript level is almost the same as in the wild type [9]. Quantification by CBB staining of samples subjected to SDS-PAGE or by immunoblotting based on total input protein revealed similar levels of both RBC-L and RBC-S between wild type and *abc4 *and that the mutant had a slightly reduced level of LHCP [9]. To address these potentially confounding factors in quantitative analysis, we developed methods to quantify transcript, protein, and metabolite levels based on genomic DNA copy number and ploidy using *A. thaliana *wild type and *abc4*.

## Results and Discussion

### Analysis of genomic DNA copy number per cell

**Figure 1:**
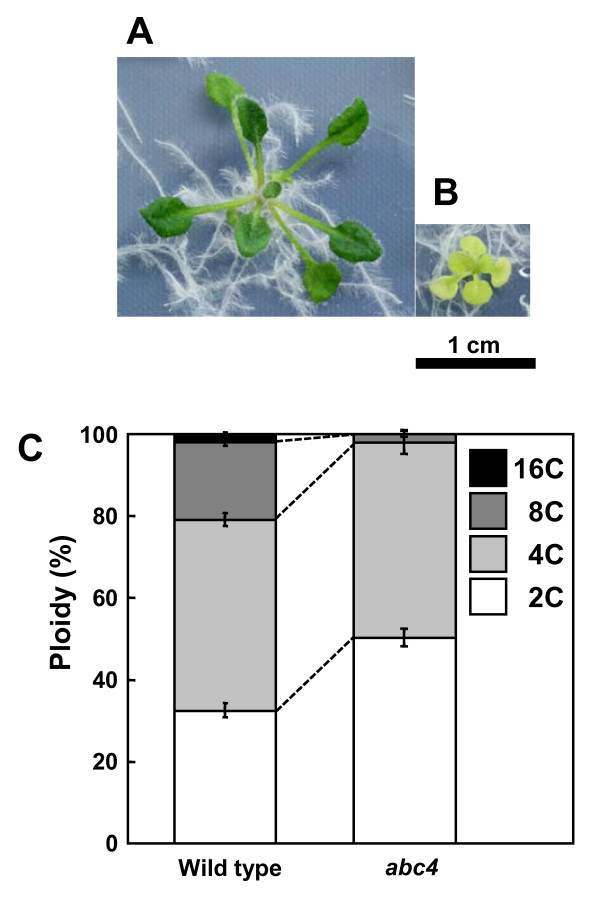
**Ploidy levels of wild-type and *abc4 *plants**. Three-week-old plants of wild type (A) and *abc4 *(B) on 1/2 MS agar medium supplemented with 1.5% sucrose. (C) Relative proportion of each cell ploidy of rosette leaves from 3-week-old plants.

Many plant cells have a unique cell cycle mode with cells undergoing iterative DNA replication without cytokinesis. This endoreduplication is frequently observed in some, but not all, plants [10]. Flow cytometry demonstrated that 32% of nuclei isolated from 3-week-old wild-type rosette leaves were in the 2C peak, and 68% of nuclei were in the 4C, 8C and 16C peaks (Figure 1A and 1C). The mutant *abc4 *had a dwarf/pale-green phenotype (Figure 1B), as expected [9]. Flow cytometry demonstrated that 50% of nuclei isolated from 3-week-old *abc4 *rosette leaves were in the 2C peak, and 50% of nuclei were in the 4C and 8C peaks (Figure 1C). Therefore, the mean ploidy of 3-week-old rosette leaves from wild type and *abc4 *was 4.35 ± 0.08 and 3.08 ± 0.03 (mean ± *s.d*.), respectively.

### Transcript accumulation normalized to genomic DNA copy number and ploidy

**Figure 2:**
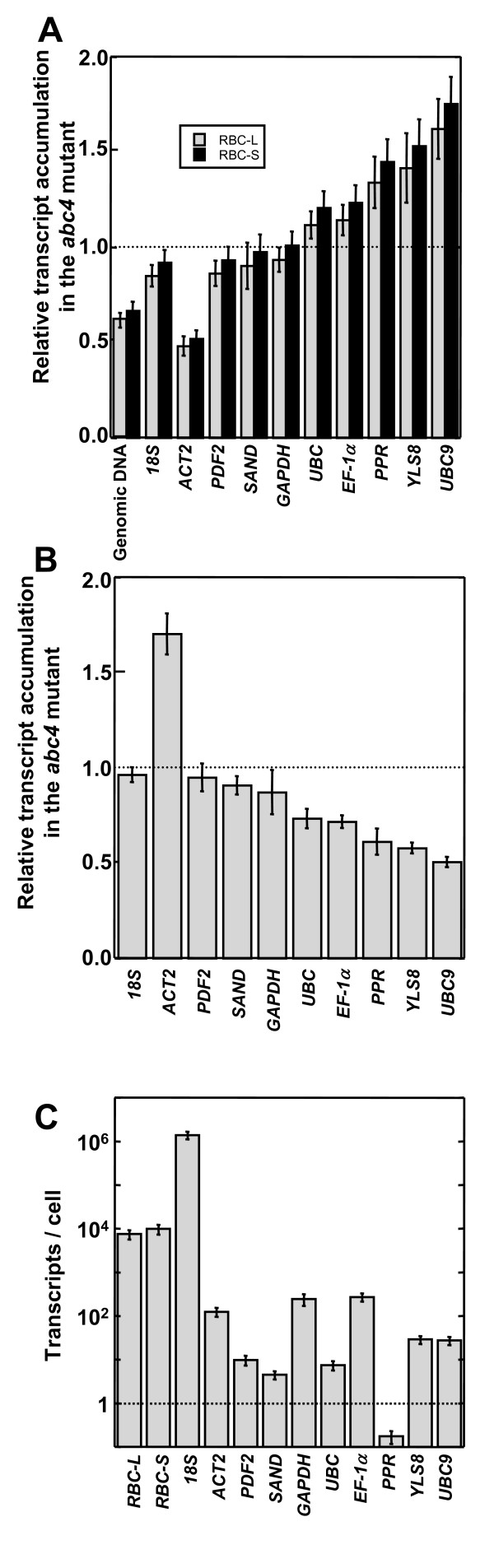
**qPCR analysis of *RBC-L *and *RBC-S *transcripts in *A. thaliana *and evaluation of reference genes**. (A) Transcript levels of *RBC-L *and *RBC-S *in *A. thaliana *rosette leaf samples were analyzed by qRT-PCR and quantified by the ΔΔCt method using the reference genes indicated at bottom. Data reflect relative transcript accumulation in *abc4 *plants relative to that in wild-type plants. (B) Transcript levels of the reference genes in rosette leaf samples from *A. thaliana *were analyzed by qRT-PCR and quantified by the ΔΔCt method using genomic DNA as the reference. Data reflect relative transcript accumulation in *abc4 *plants relative to that in wild-type plants. (C) Transcripts numbers per cell in the wild-type *A. thaliana *rosette leaf were determined relative to genomic DNA using qPCR analysis. Data reflect the mean ± *s.d*. from duplicate experiments of four biological samples.

We used qRT-PCR to compare transcript levels of *RBC-L *and *RBC-S *between wild-type and *abc4 *plants. For quantification using the ΔΔCt method [11], one of several established housekeeping genes, namely *ACT2 *(actin 2), *PDF2 *(transposable element gene), *SAND *(SAND family protein), *GAPDH *(glyceraldehyde 3-phosphate dehydrogenase), *UBC *(ubiquitin-conjugating enzyme), *EF-1α *(elongation factor 1-*α*), *PPR *(pentatricopeptide repeat-containing protein), *YLS8 *(yellow-leaf-specific protein 8), *UBC9 *(ubiquitin-conjugation enzyme E2), or the *18S *rRNA gene or genomic DNA (Figure 2A), was used as the reference (see additional file 1). Derivation of the 2^-ΔΔCt ^equation, including assumptions, experimental design, and validation tests, is described in the Applied Biosystems User Bulletin # 2 http://www3.appliedbiosystems.com/cms/groups/mcb_support/documents/generaldocuments/cms_040980.pdf. When genomic DNA was used as reference, the relative transcript accumulation was calculated as follows: $2^{-\Delta \Delta Ct}\times \frac{G_{mutant}}{G_{wild}}=2^{-\Delta \Delta Ct}\times \frac{3.08}{4.35}$, where *G_mutant _*and *G_wild _*are the genomic DNA copy number per cell (i.e., mean ploidy) of the *abc4 *and wild-type plants, respectively (see additional file 2). Using *ACT2 *as the reference, the *RBC-L and RBC-S *transcripts were lower in *abc4 *plants (Figure 2A; also see additional file 3). By contrast, the levels were comparable between wild type and mutant using *SAND *or *GAPDH *as the reference, and the levels were higher in the mutant than in the wild type using *UBC, EF-1α, PPR, YLS8 *or *UBC9 *as the reference. Use of genomic DNA, *18S *rRNA, or *PDF2 *as a reference revealed slightly lower levels of both *RBC-L *and *RBC*-*S *transcripts in the mutant. Figure 2B shows the transcript levels of the genes often used as references in *abc4 *relative to the wild type using genomic DNA as the reference. This analysis indicated that the *18S *and *PDF2 *transcript levels were similar between the wild type and mutant. The level of *ACT2 *transcript was significantly higher in the mutant, whereas levels of *SAND, GAPDH, UBC, EF-1α, PPR, YLS8 *and *UBC9 *transcripts were lower in the mutant. We concluded that differences between the transcript levels of the reference genes in the wild type and mutant (Figure 2B) resulted in apparent differences in *RBC-L *and *RBC-S *transcript levels between the wild type and mutant (Figure 2A). In this assay, the wild type and mutant had comparable levels of *18S *transcript, but *18S *expression is not always consistent between cells [7,8].

Northern hybridization using total RNA revealed that the *RBC-L *and *RBC*-*S *transcript levels were significantly elevated in *abc4 *[9]. Note that qRT-PCR normalized to the genes often used as references may provide misleading results (Figure 2A). However, qRT-PCR using genomic DNA copy number and the mean ploidy as the reference can provide more accurate information on the level of transcripts per cell.

### Quantification of transcript number per cell

In 3-week-old wild-type plants, we analyzed the number of transcripts by qRT-PCR using genomic DNA as reference (Figure 2C). We determined the DNA copy number by qRT-PCR and calculated the transcript number per cell as follows:

$$\begin{array}{l} \left(\text{transcript number}/\text{cell}\right)\\ =(\text{transcript number}/\text{genomic DNA copy number})\times (\text{genomic DNA copy number/cell})\\ =2^{-\Delta \text{Ct}}\times 4.35\\ =2^{-\{\text{Ct}(\text{cDNA})-\text{Ct}(\text{genome})\}}\times 4.35 \end{array}$$

Each cell in the wild-type rosette leaf had an average of 7.5 × 10^3 ^*RBC-L *transcripts, 9.9 × 10^3 ^*RBC-S *transcripts, and 1.4 × 10^6 ^*18S rRNA *transcripts (see additional file 4). The number of *PPR*/At1g62930 (pentatricopeptide repeat-containing protein gene) transcripts per cell was much lower (0.17) (Figure 2C; also see additional file 4), indicating that there is less than one *PPR *transcript per cell. *PPR *was not transcribed in all cells of wild-type rosette leaves. The rosette leaf contains various cell types (e.g., mesophyll cells, epidermal cells, guard cells and vascular tissue cells), and *PPR *transcription may be cell type specific. Of course, any gene with less than one transcript per cell should not be used as a reference for quantification of qRT-PCR data.

### Protein accumulation normalized to genomic DNA copy number and ploidy

**Figure 3:**
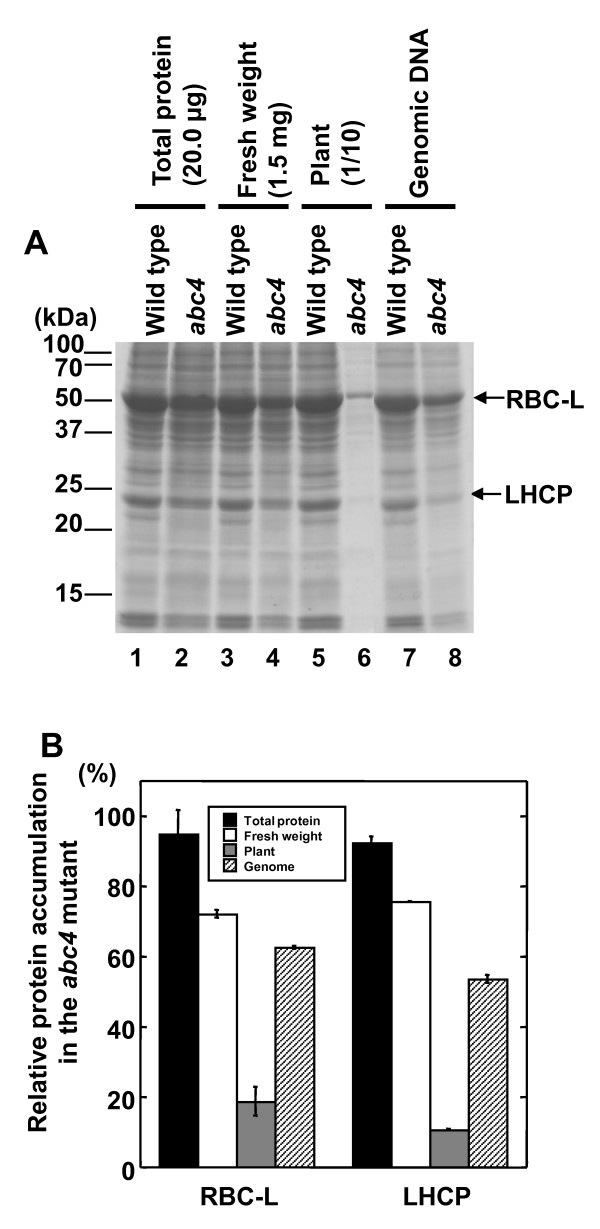
**Analysis of RBC-L and LHCP accumulation in *A. thaliana***. (A) Total protein extract prepared from 3-week-old wild-type (odd-numbered lanes) and *abc4 *(even-numbered lanes) plants was subjected to SDS-PAGE analysis followed by CBB staining; sample amounts varied according to the normalization factor indicated at top. Lanes 1 and 2, 20.0 μg total protein; lanes 3 (17.9 μg total protein) and 4 (13.5 μg total protein) correspond to 1.5 mg of fresh weight; lanes 5 (18.9 μg total protein) and 6 (1.2 μg total protein) correspond to 10% of the material from an individual plant (wild type: 1.55 mg of fresh weight; *abc4*: 0.35 mg of fresh weight); lanes 7 (20.0 μg total protein) and 8 (12.4 μg total protein) correspond to equivalent cell numbers of wild-type and mutant samples. (B) Quantification of RBC-L and LHCP accumulation in wild-type and *abc4 *plants. The density of each CBB-stained protein band in panel (A) was quantified using ImageJ http://rsbweb.nih.gov/ij/. Data reflect the mean ± standard deviation (*n *= 4) relative to the indicated normalization factor.

We analyzed protein expression in 3-week-old wild-type and *abc4 *rosette leaves by SDS-PAGE and quantified CBB-stained RBC-L and LHCP bands using ImageJ http://rsbweb.nih.gov/ij/ based on several normalization criteria. When the mass of the total input protein was used for normalization, the RBC-L and LHCP band intensities were nearly identical between wild-type and *abc4 *samples (Figure 3A lanes 1 and 2, and Figure 3B), but the amounts of these proteins were slightly lower in *abc4 *when fresh weight was used for normalization (Figure 3A lanes 3 and 4, and Figure 3B). The fresh weight of all rosette leaves from individual 3-week-old wild-type and *abc4 *plants was 15.5 ± 0.3 mg and 3.5 ± 0.1 mg, respectively (mean ± *s.e.m*., *n *= 20 for both). Based on the individual mean fresh weight of rosette leaves, the amount of RBC-L and LHCP protein in *abc4 *was significantly lower than in wild type (Figure 3A lanes 5 and 6, and Figure 3B).

We next quantified protein expression levels normalized to genomic DNA copy number and ploidy established by qPCR using plant extracts. To extract total protein, the plants were homogenized in an extraction buffer containing 10 mM EDTA and 1.0% SDS, both of which inhibit DNase (see Methods) [12]. Because the KAPA2G Robust HotStart DNA polymerase (Kapa Biosystems) used in this assay is tolerant to a wide range of common PCR inhibitors (e.g., salts and SDS), the protein samples were used without further purification as the qPCR template. Protein samples from equivalent numbers of wild type and mutant cells were then subjected to SDS-PAGE; for this analysis the volume of protein sample from the mutant (*V_mutant_*) was applied. $V_{mutant}=V_{wild}\times \frac{1}{R}\times \frac{G_{mutant}}{G_{wild}}=V_{wild}\times \frac{1}{R}\times \frac{3.08}{4.35}$, where *V_wild _*is the applied volume of protein sample from wild type, *R *is the relative genomic DNA copy number of the protein sample from the mutant to that of wild type, and *G_mutant _*and *G_wild _*are the genomic DNA copy number per cell (i.e., mean ploidy) of the *abc4 *and wild-type plants, respectively (see Methods and additional file 5). The amounts of RBC-L and LHCP in *abc4 *were significantly lower than in the wild type based on genomic DNA copy number (Figure 3A lanes 7 and 8, and Figure 3B).

### Quantification of protein and metabolite number per cell

**Figure 4:**
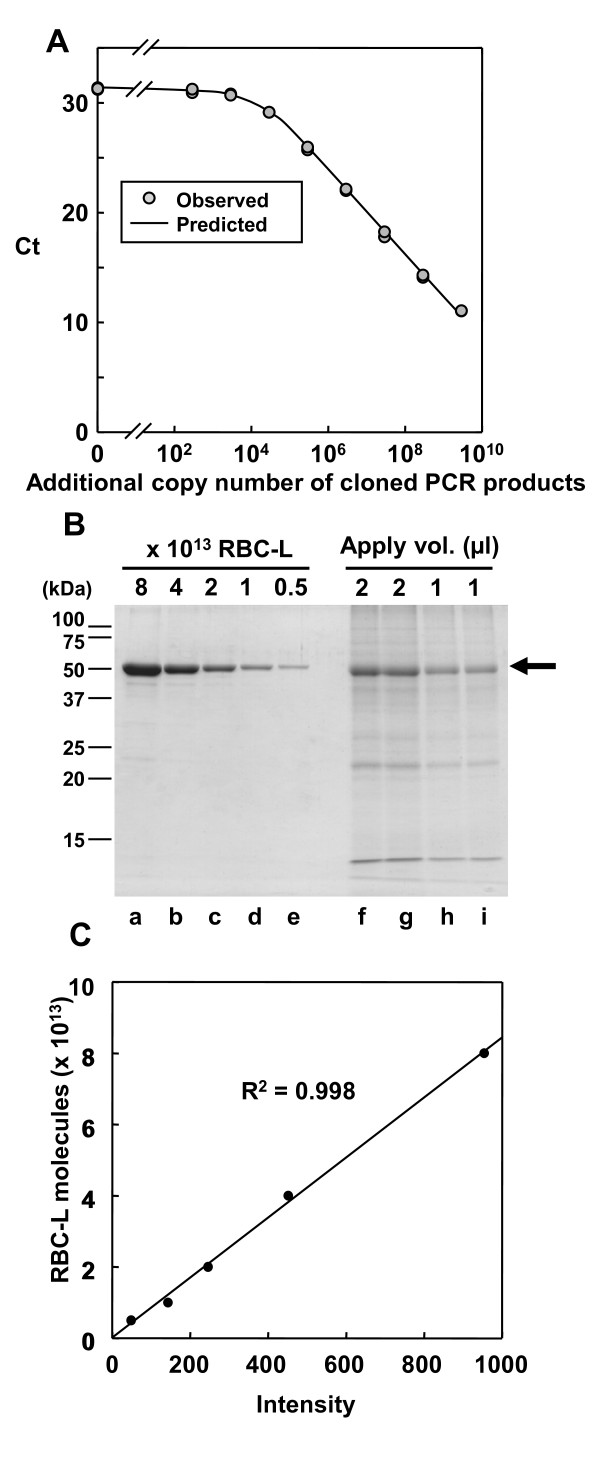
**Quantification of RBC-L protein and genomic copy numbers in protein extracts from *A. thaliana***. (A) Standard curve indicating Ct as a function of the copy number of the cloned PCR product that was added to PCR reactions containing 1 μl of a 1:40 dilution of wild-type protein extract. Shown in the graph is an example of observed Ct values as a function of exogenously added DNA molecules. Each reaction contained 1 μl of a 1:40 dilution of wild-type protein extract and an arbitrary amount of external plasmid (p) harboring DNA fragments amplified by PCR using the primers T7F6-3-F and T7F6-3-R (see additional file 1). (B) SDS-PAGE analysis of RBC-L accumulation in *A. thaliana*. The indicated number of purified recombinant RBC-L molecules (lanes a to e) and the indicated amount of wild-type protein extract sample (lanes f to i) was loaded, and the gel was stained with CBB. The arrow indicates RBC-L migration. (C) Standard curve indicating the number of recombinant RBC-L molecules relative to the intensity of CBB staining.

We next analyzed the number of genomic DNA molecules and RBC-L molecules per cell in protein samples of 3-week-old wild-type rosette leaves. One microliter of a 1:40 dilution of wild-type protein extract and 1 μl of plasmid harboring a DNA fragment amplified by PCR (between 2.88 × 10^2 ^and 2.88 × 10^9 ^molecules, or 0 molecules) were added to the PCR reactions. This mixing procedure was done because the amplification efficiency of the qPCR differed depending on whether the protein extract or the purified plasmid was used as template (efficiency = 0.665 or 0.890, respectively). Therefore, we could not extrapolate the genomic DNA copy number in the protein samples using the standard curve created with the purified plasmid. Thus, to estimate genomic DNA copy number, we assessed the effect of exogenously added plasmid DNA in PCR reactions containing genomic DNA on PCR amplification of a DNA segment. This process can be formulated as follows:

(*g *+ *p*)(2*E*)*^Ct ^*= *A*, where *g *is the genomic DNA copy number, *p *is the plasmid copy number, *Ct *is the PCR cycle number, *E *is the PCR amplification coefficient, and *A *is the number of amplified molecules. The nonlinear least-squares method was used to obtain the parameters *g*, *E *and *A *(R language; http://www.r-project.org) (see additional file 6). The qPCR analysis yielded the genomic DNA copy number in 1 μl of the protein sample from the wild-type plant (Figure 4A, Table 1). SDS-PAGE analysis followed by CBB staining was used to determine the number of RBC-L molecules in 1 μl of the wild-type protein sample (Figure 4B, Figure 4C and Table 1); recombinant RBC-L purified from *Escherichia coli *was used as a control. These results indicated that the wild-type protein sample had 2.63 × 10^8 ^± 0.15 × 10^8 ^(mean ± *s.d., n *= 4) molecules of RBC-L per cell. In plants, Rubisco consists of eight large and eight small subunits [13]. Therefore, each cell had 3.29 × 10^7 ^Rubisco complexes. Furthermore, we measured the chlorophyll content [14] in the protein sample from 3-week-old wild-type rosette leaves (Table 1). The molecular weights of chlorophyll *a *and chlorophyll *b *are 893 and 907, respectively. We determined that 1 μl of the wild-type protein sample had between 9.88 × 10^13 ^and 1.98 × 10^14 ^chlorophyll molecules, indicating that each cell had 1.85 × 10^9 ^± 0.09 × 10^9 ^(mean ± *s.d., n = *4) molecules of chlorophyll. The fact that the coefficient of variation for the number of RCB-L and chlorophyll molecules per genome was relatively low (5.70 × 10^-2 ^and 4.86 × 10^-2^, respectively; Table 1) suggested that quantification based on the genomic DNA copy number was reproducible.

**Table 1:** Numbers of molecules in 3-week-old rosette leaves of wild-type Arabidopsis.

Sample numbers	#1	#2	#3	#4
Genomic copy number/μl	2.47 × 10^5^	2.40 × 10^5^	4.12 × 10^5^	4.39 × 10^5^
*s.e.m*	0.16 × 10^5^	0.22 × 10^5^	0.47 × 10^5^	0.33 × 10^5^
n	11	10	20	20

RBC-L number/μl	1.48 × 10^13^	1.57 × 10^13^	2.40 × 10^13^	2.53 × 10^13^
*s.d*.	0.05 × 10^13^	0.18 × 10^13^	0.36 × 10^13^	0.18 × 10^13^
n	4	4	4	4

^1^RBC-L number/genome	5.99 × 10^7^	6.54 × 10^7^	5.83 × 10^7^	5.76 × 10^7^

^2^RBC-L number/cell	2.60 × 10^8^	2.84 × 10^8^	2.54 × 10^8^	2.51 × 10^8^

**^3^RBC-L number/cell**	**2.63 × 10^8^**			
***s.d*.**	**0.15 × 10^8^**			
**^4^CV**	**5.70 × 10^-2^**			

Chlorophyll number/μl	9.88 × 10^13^	10.01 × 10^13^	17.72 × 10^13^	19.78 × 10^13^
*s.d*.	0.81 × 10^13^	0.20 × 10^13^	0.15 × 10^13^	0.04 × 10^13^
n	2	2	2	2

^5^Chlorophyll number/genome	4.00 × 10^8^	4.17 × 10^8^	4.30 × 10^8^	4.51 × 10^8^

^6^Chlorophyll number/cell	1.74 × 10^9^	1.81 × 10^9^	1.84 × 10^9^	1.96 × 10^9^

**^3^Chlorophyll number/cell**	**1.85 × 10^9^**			
***s.d*.**	**0.09 × 10^9^**			
**^4^CV**	**4.86 × 10^-2^**			

^1^(RBC-L number/genome) = (RBC-L number/μl)/(Genome copy number/μl)

^2^(RBC-L number/cell) = (RBC-L number/genome) × (Genome copy number/cell)

= (RBC-L number/genome) × 4.35

^3^Mean of sample number 1 to 4

^4^Coefficient of variation (*s.d*./mean)

^5^(Chlorophyll number/genome) = (Chlorophyll number/μl)/(Genome copy number/μl)

^6^(Chlorophyll number/cell) = (Chlorophyll number/genome) × (Genome copy number/cell)

= (Chlorophyll number/genome) × 4.35

We quantified the amount of RBC-L according to the intensity of CBB staining following SDS/PAGE analysis. However, it is also possible to use immunoblotting or enzyme-linked immunosorbent assay to quantify the number of molecules of a particular protein per genomic DNA copy number.

## Conclusions

In this study, we demonstrate that establishing an appropriate normalization factor is a challenging yet vital aspect of comparing protein, transcript, or metabolite levels among samples. Our results establish a facile and accurate method for quantifying these molecules based on genomic DNA copy number and ploidy. Although we performed cytometric analysis to measure the ploidy, a simplified method without the cytometric analysis may be applicable when assessing the effect(s) of a short-term treatment (e.g., induction of stress over several hours). Furthermore, our method can provide information on the number of transcripts, proteins, and metabolites per cell, and it should be applicable for stoichiometry and mathematical modeling of cellular systems.

## Methods

### Plant materials

*Arabidopsis thaliana*, Wassilewskija (wild type) and *abc4 *[9] (Wassilewskija background) were grown at 23°C under continuous light (32.0 μmol·m^-2^s^-1^) on plates containing 1/2 MS medium and 1.5% sucrose.

### Ploidy measurement

Flow cytometry was performed by a Ploidy Analyzer (Partec, Münster, Germany) [15]. At least 5,000 nuclei isolated from rosette leaves of each 3-week-old Arabidopsis plant were used for each ploidy measurement. Three biological and two technical replicates were used for each sample analyzed.

### Preparation of total nucleic acid from *A. thaliana*

Rosette leaves (100 mg) from each 3-week-old Arabidopsis plant were ground with a mortar and pestle in liquid nitrogen and homogenized in 5 volumes (v/w) of extraction buffer (100 mM MOPS-KOH, pH 7.0, 10 mM EDTA, 0.3 M NaCl, 1.0% SDS). Extraction of total nucleic acids (genomic DNA and total RNA) was performed by the addition of 5 volumes of phenol saturated with 1 M MOPS-KOH, pH 7.0, and 5 volumes of chloroform:isoamyl alcohol (24:1, v/v) followed by vigorous agitation and centrifugation for 10 min at 20,000 × *g*. The aqueous phase was collected and extracted two times with an equal volume of phenol:chloroform:isoamyl alcohol (25:24:1, v/v/v). The nucleic acids were precipitated with ethanol and suspended in 50 μl nuclease-free water (see additional file 7).

### Preparation of cDNA and genomic DNA

To prepare cDNAs, 4 μl total nucleic acid (A_260 _= 20.0) was digested with Turbo *DNA-free *DNase I (Ambion, Austin, TX, USA) according to the manufacturer's instructions. Absence of genomic DNA in DNase I treated samples was verified by PCR using primers T7F6-F-2 and T7F6-R-2 or MDC16-F-2 and MDC16-R-2 (see additional file 1). cDNA synthesis was performed using the PrimeScript RT reagent kit in the presence of oligo dT and random 6-mer primers according to the manufacturer's instructions (Takara Bio. Inc., Ohtsu, Japan). To prepare genomic DNA, 4 μl total nucleic acid (A_260 _= 20.0) was digested with RNase (Wako Pure Chemical Industries, Osaka, Japan) (see additional files 7 and 8).

### Protein sample preparation

Rosette leaves (100 mg) from each 3-week-old plant were ground with a mortar and pestle in liquid nitrogen and transferred to a new 2-ml tube and homogenized in 5 volumes (v/w) of extraction buffer containing 15 mM Tris-HCl, pH 8.0, 50 mM NaCl, 10 mM EDTA, 1.0% SDS and 1.0% protease inhibitor cocktail (Sigma-Aldrich, Tokyo, Japan). The samples were incubated on ice for 10 min with vigorous vortexing every minute. The samples were centrifuged for 5 min at 10,000 × *g*, and the supernatants were used for further experiments. The total protein concentration of each supernatant was determined using a Coomassie Protein Assay kit (Pierce Biotechnology, Rockford, IL, USA). Chlorophyll concentrations were calculated according to Arnon [14].

### qPCR analysis

All qPCR reactions were analyzed with an ABI PRISM 7300 sequence detection system (Applied Biosystems, Foster City, CA, USA), and data were analyzed using SDS 2.2.1 software (Applied Biosystems). The KAPA SYBR FAST qPCR kit (Kapa Biosystems, Boston, MA, USA) was used for qPCR amplification of purified genomic DNA or cDNA according to the manufacturer's instructions; the 20-μl reactions contained 1 μl of genomic DNA or cDNA template and 8 pmol of each set of gene-specific primers (see additional files 1). PCR reaction conditions were as follows: 95°C for 3 min, followed by 40 cycles of 95°C for 15 s and 60°C for 40 s. Transcript levels were quantified by the ΔΔCt method [11]. The KAPA2G Robust HotStart kit (Kapa Biosystems) was used for qPCR amplification of genomic DNA in protein extract supernatants; the 20-μl reactions contained 4 μl of undiluted KAPA2G Buffer B, 4 μl of undiluted Enhancer 1, 1.6 μl of 2.5 mM dNTP, 8 pmol of each gene-specific primer (T7F6-3-F and T7F6-3-R, see additional file 1), 1 μl of 0.1% SYBR Green I (Takara), 0.4 μl Rox High (from the KAPA SYBR FAST qPCR kit), 2 U of KAPA2G Robust HotStart DNA polymerase, and 1 μl of protein extract. For quantification of genomic DNA copy number in the mutant plants relative to wild-type plants, 1 μl of protein extract of wild-type plants (diluted 1:10, 1:20, 1:40, 1:80 or 1:160) was added to the PCR reactions, and a standard curve was generated by qPCR (see additional file 5). One microliter of a 1:40 dilution of protein extract of the mutant plants was added to the PCR reactions, and qPCR was performed. For absolute quantification of genomic DNA copy number in protein extracts of wild-type plants, 1 μl of a 1:40 dilution of wild-type protein extract and 1 μl of cloned PCR products (between 2.88 × 10^2 ^and 2.88 × 10^9 ^molecules, or 0 molecules) was added to the PCR reactions; the 20-μl reactions contained 4 μl of undiluted KAPA2G Buffer B, 4 μl of undiluted Enhancer 1, 4.4 μl of 25 mM MgCl_2_, 1.6 μl of 2.5 mM dNTP, 8 pmol of each gene-specific primer (T7F6-3-F and T7F6-3-R, see additional file 1), 10 pmol of TaqMan Probe (see additional file 1), 0.4 μl Rox High (from the KAPA SYBR FAST qPCR kit), 2 U of KAPA2G Robust HotStart DNA polymerase, and 1 μl of protein extract, and PCR was performed. This dilution series was prepared with EASY Dilution (for real-time PCR) (Takara). PCR reaction conditions were as follows: 95°C for 3 min, followed by 40 cycles of 95°C for 15 s and 72°C for 40 s.

### Preparation of cloned PCR products

PCR was performed using primers T7F6-3-F and T7F6-3-R (see additional file 1) with genomic DNA of wild-type Arabidopsis as template. The amplified DNA fragment was ligated into the TA cloning vector, pMD20 (Takara), and the sequence was confirmed. The plasmids containing the PCR products were digested with *Xho*I and purified. The mass of a nucleotide pair in DNA is 660 Da, and the plasmid containing the PCR product was 2,872 bp. The concentration of the linearized plasmid was determined, and the number of plasmid molecules was calculated.

### Expression and purification of RBC-L

The full-length open reading frame of RBC-L was amplified by PCR using primers containing an *Nde*I site (RBCL-Nde, 5'-CCCCATATGTCACCACAAACAGAGACTAAAG-3'; *Nde*I site underlined) and a *Xho*I site (RBCL2-Xho, 5'-CCCCTCGAGCTCTTGGCCATCTAATTTATCGATG-3'; *Xho*I site underlined). The amplified DNA fragment was digested with *Nde*I and *Xho*I and ligated into the expression vector pET-24a(+) (Novagen, San Diego, CA, USA). RBC-L was expressed and purified as described [16]. The molecular weight of RBC-L was 54,019. The concentration of the purified RBC-L was determined, and the number of RBC-L molecules was calculated.

## List of abbreviations

LHCP: light-harvesting chlorophyll *a/b *protein; qPCR: quantitative PCR; qRT-PCR: quantitative reverse transcription PCR; RBC or Rubisco: ribulose 1,5-bisphosphate carboxylase/oxygenase; RBC-L: large subunit of Rubisco; RBC-S: small subunit of Rubisco; *ACT2*: actin 2; *PDF2*: transposable element gene; *SAND*: SAND family protein; *GAPDH*: glyceraldehyde 3-phosphate dehydrogenase; *UBC*: ubiquitin-conjugating enzyme; *EF-1α*: elongation factor 1-*α*; *PPR*: pentatricopeptide repeat-containing protein; *YLS8*: yellow-leaf-specific protein 8; *UBC9*: ubiquitin-conjugation enzyme E2

## Competing interests

The authors declare that they have no competing interests.

## Authors' contributions

HS conceived and directed this study, performed all experimental work, and wrote the paper with input from co-authors. TO calculated the copy number of genomic DNA in protein samples. NT and MM performed the flow cytometry analysis. AS contributed to data analysis and provided advice. All authors read and approved the final manuscript.

## Supplementary Material

Additional File 1 **Primers used in this work**. Table shows the primer names and the sequences. Click here for file

Additional File 2 **qPCR amplification and dissociation curves**. (A) Schematic representation of each PCR-amplified region in chromosomes I-V (see additional file 1). (B) Real-time qPCR amplification curves generated using equal volume of template and the primer sets indicated. The curves using the different primer sets were the same, suggesting that the amplification efficiency using three different primer sets and the extraction efficiency of the different regions of genomic DNA were essentially equivalent. (C) Dissociation curves for the PCR products generated using the indicated primer sets. The *y *axis shows the logarithm of fluorescence. These curves reflect normalized data. Click here for file

Additional File 3 **Dissociation curves**. Dissociation curves for the PCR products generated using the primer set of T7F6-F-2 and T7F6-R-2 (A), MDC16-F-2 and MDC16-R-2 (B), 18S-3-F and 18S-3-R (C), RBCL-2-F and RBCL-2-R (D), or RBCS-3-F and RBCS-3-R (E) (see additional file 1). The *y *axis shows the logarithm of fluorescence. These curves reflect normalized data. Click here for file

Additional File 4 **Transcript number per cell in 3-week-old rosette leaves of wild-type Arabidopsis**. Table shows the transcript number of *RBC-L*, *RBC-S*, *18S*, *ACT2*, *PDF2*, *SAND*, *GAPDH*, *UBC*, *EF-1α*, *PPR*, *YLS8 *and *UBC9 *genes per cell. Click here for file

Additional File 5 **Standard curve indicating the Ct relative the dilution of protein extract sample from a wild-type plant as template for qPCR**. One microliter of wild-type protein extract (diluted 1:10, 1:20, 1:40, 1:80, or 1:160) was used as template for qPCR. Click here for file

Additional File 6 **Calculation of genomic DNA copy number in protein extracts**. This file shows mathematical formula with actual command sequences on R language to calculate genomic DNA copy number. Click here for file

Additional File 7 **Scheme for preparing cDNA and genomic DNA**. Scheme shows the methods of extraction of the nucleic acid (genomic DNA and total RNA), DNase I digestion, reverse transcription and RNase digestion, and the extraction buffer composition. Click here for file

Additional File 8 **Agarose gel analysis of total nucleic acid, genomic DNA, and total RNA preparations**. Total nucleic acid from wild-type plants was treated without (lane 1) or with RNase (lane 2) or DNase (lane 3) and subjected to 1.2% agarose gel electrophoresis followed by ethidium bromide staining. Click here for file
