# Supplementary material for: Normalization using ploidy and genomic DNA copy number allows absolute quantification of transcripts, proteins and metabolites in cells
Source: Plant Methods. 2010 Dec 29;6:29. doi: 10.1186/1746-4811-6-29 (PMC3023742; doi:10.1186/1746-4811-6-29)
Supplement: Additional File 1 — Primers used in this work. Table shows the primer names and the sequences. [file 1746-4811-6-29-S1.PDF]

Additional File 1   Primers using this work

Name	Sequence (5' → 3')	Gene ID	Source
T7F6-F-2	AAAAACTCTGACCCGGTCGCACCTGAAGTC		this study
T7F6-R-2	GCCGGAGCTTACGGGAACAAATGGTGACTA		
T7F6-3-F	AAAAACTCTGACCCGGTCGCACCTGAA		this study
T7F6-3-R	GCCGGAGCTTACGGGAACAAATGGT		
TaqMan	FAM-ACGGTGGGGTTCTCTCTCTC-TAMRA		this study
MDC16-F-2	CCACAAGAGATTGGCGACGATGAGGATGAT		this study
MDC16-R-2	AGATCACGGCCTTTTTCTTCCCACCACGTA		
MUG13-F-2	TGAAGAAGGCAGGAAGACCGTCAGAACGAA		this study
MUG13-R-2	ATGAAGATGCACGCACTCGTGTTGCATAGG		
18S-3-F	GCCCGGGTAATCTTTGAAAT	At3g41768	this study
18S-3-R	GTACAAAGGGCAGGGACGTA	At2g01010	
RBCL-2-F	TTCGGTGGAGGAACTTTAGG	ArthCp030	this study
RBCL-2-R	GCAAGATCACGTCCCTCATT		
RBCS-3-F	GGATTTGTGTACCGTGAGCA	At1g67090, At5g38430	this study
RBCS-3-R	ACTTGAGCGGAGTCGGTGCA	At5g38420, At5g38410	
ACT2-F	CTTGCACCAAGCAGCATGAA	At3g18780	Reference 1
ACT2-R	CCGATCCAGACACTGTACTTCCTT		
UBC-F	CTGCGACTCAGGGAATCTTCTAA	At5g25760	Reference 1
UBC-R	TTGTGCCATTGAATTGAACCC		
EF-1α-F	TGAGCACGCTCTTCTTGCTTTCA	At5g60390	Reference 1
EF-1α-R	GGTGGTGGCATCCATCTTGTTACA		
GAPDH-F	TTGGTGACAACAGGTCAAGCA	At1g13440	Reference 1
GAPDH-R	AAACTTGTCGCTCAATGCAATC		
PDF2-F	TAACGTGGCCAAAATGATGC	At1g13320	Reference 1
PDF2-R	GTTCTCCACAACCGCTTGGT		
PPR-F	GAGTTGCGGGTTTGTTGGAG	At1g62930	Reference 1
PPR-R	CAAGACAGCATTTCCAGATAGCAT		
SAND-F	AACTCTATGCAGCATTTGATCCACT	At2g28390	Reference 1
SAND-R	TGATTGCATATCTTTATCGCCATC		
UBC9-F	TCACAATTTCCAAGGTGCTGC	At4g27960	Reference 1
UBC9-R	TCATCTGGGTTTGGATCCGT		
YLS8-F	TTACTGTTTCGGTTGTTCTCCATTT	At5g08290	Reference 1
YLS8-R	CACTGAATCATGTTCGAAGCAAGT		

Reference

1 Czechowski T. Stitt M, Altmann T, Udvardi MK, Scheible WR: **Genome-wide identification and testing of superior reference genes for transcript normalization in Arabidopsis.** *Plant Physiol* 2005, **139**: 5-17.
