# Supplementary material for: Normalization using ploidy and genomic DNA copy number allows absolute quantification of transcripts, proteins and metabolites in cells
Source: Plant Methods. 2010 Dec 29;6:29. doi: 10.1186/1746-4811-6-29 (PMC3023742; doi:10.1186/1746-4811-6-29)
Supplement: Additional File 2 — qPCR amplification and dissociation curves. (A) Schematic representation of each PCR-amplified region in chromosomes I-V (see additional file 1). (B) Real-time qPCR amplification curves generated using equal volume of template and the primer sets indicated. The curves using the different primer sets were the same, suggesting that the amplification efficiency using three different primer sets and the extraction efficiency of the different regions of genomic DNA were essentially equivalent. (C) Dissociation curves for the PCR products generated using the indicated primer sets. The y axis shows the logarithm of fluorescence. These curves reflect normalized data. [file 1746-4811-6-29-S2.PDF]

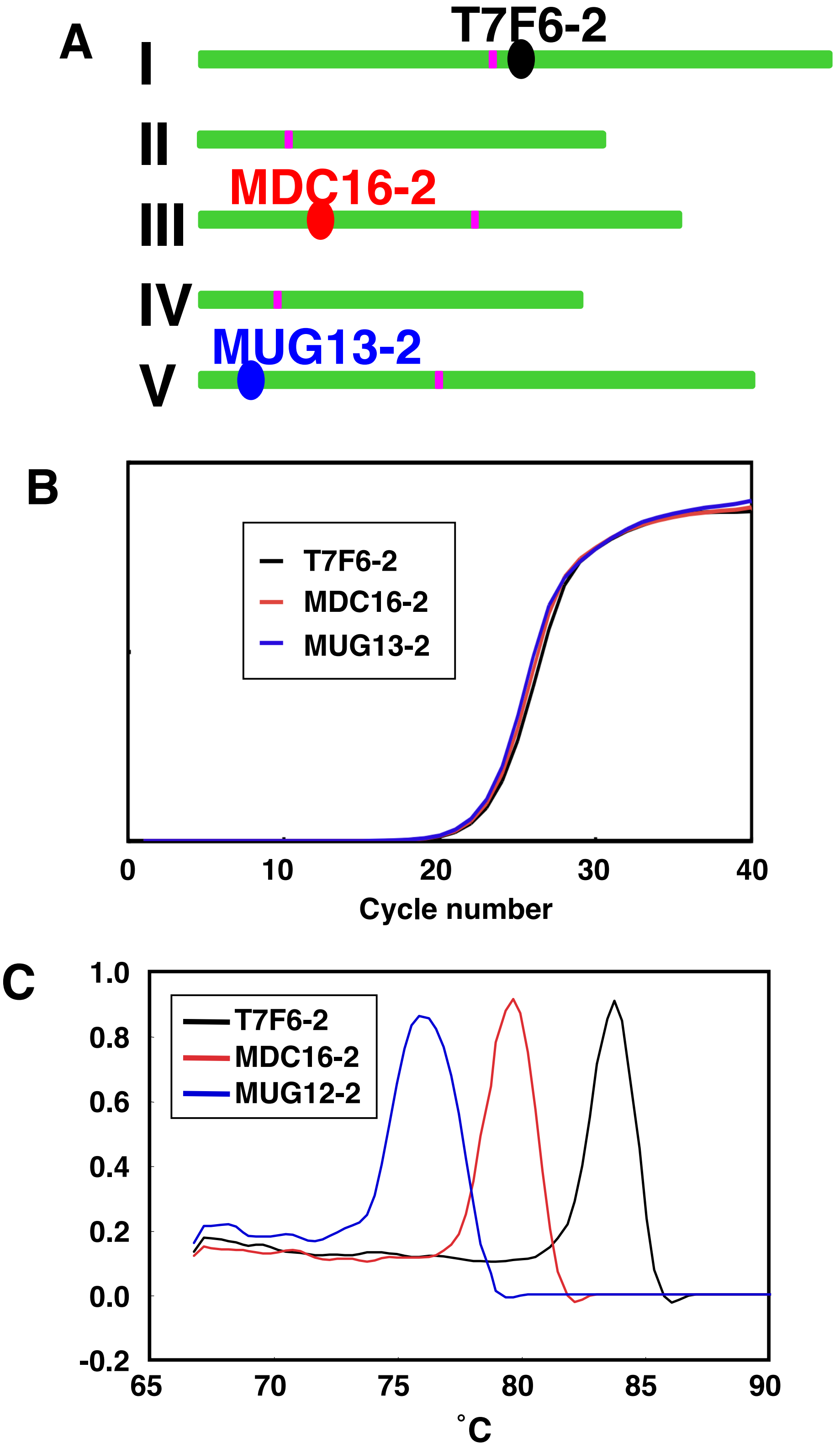

Additional File 2 qPCR amplification and dissociation curves. (A) Schematic representation of each PCR-amplified region in the chromosomes I-V (see additional file 1). (B) Real-time qPCR amplification curves generated using equal volume of template and the primer sets indicated. The curves using the different primer sets were the same, suggesting that the amplification efficiency using three different primer sets and the extraction efficiency of the different regions of genomic DNA were essentially equiavalent. (C) Dissociation curves for the PCR products generated using the indicated primer sets. The *y* axis shows the logarithmic fluorescence. These curves reflect normalized data.
