# Supplementary material for: Normalization using ploidy and genomic DNA copy number allows absolute quantification of transcripts, proteins and metabolites in cells
Source: Plant Methods. 2010 Dec 29;6:29. doi: 10.1186/1746-4811-6-29 (PMC3023742; doi:10.1186/1746-4811-6-29)
Supplement: Additional File 3 — Dissociation curves. Dissociation curves for the PCR products generated using the primer set of T7F6-F-2 and T7F6-R-2 (A), MDC16-F-2 and MDC16-R-2 (B), 18S-3-F and 18S-3-R (C), RBCL-2-F and RBCL-2-R (D), or RBCS-3-F and RBCS-3-R (E) (see additional file 1). The y axis shows the logarithm of fluorescence. These curves reflect normalized data. [file 1746-4811-6-29-S3.PDF]

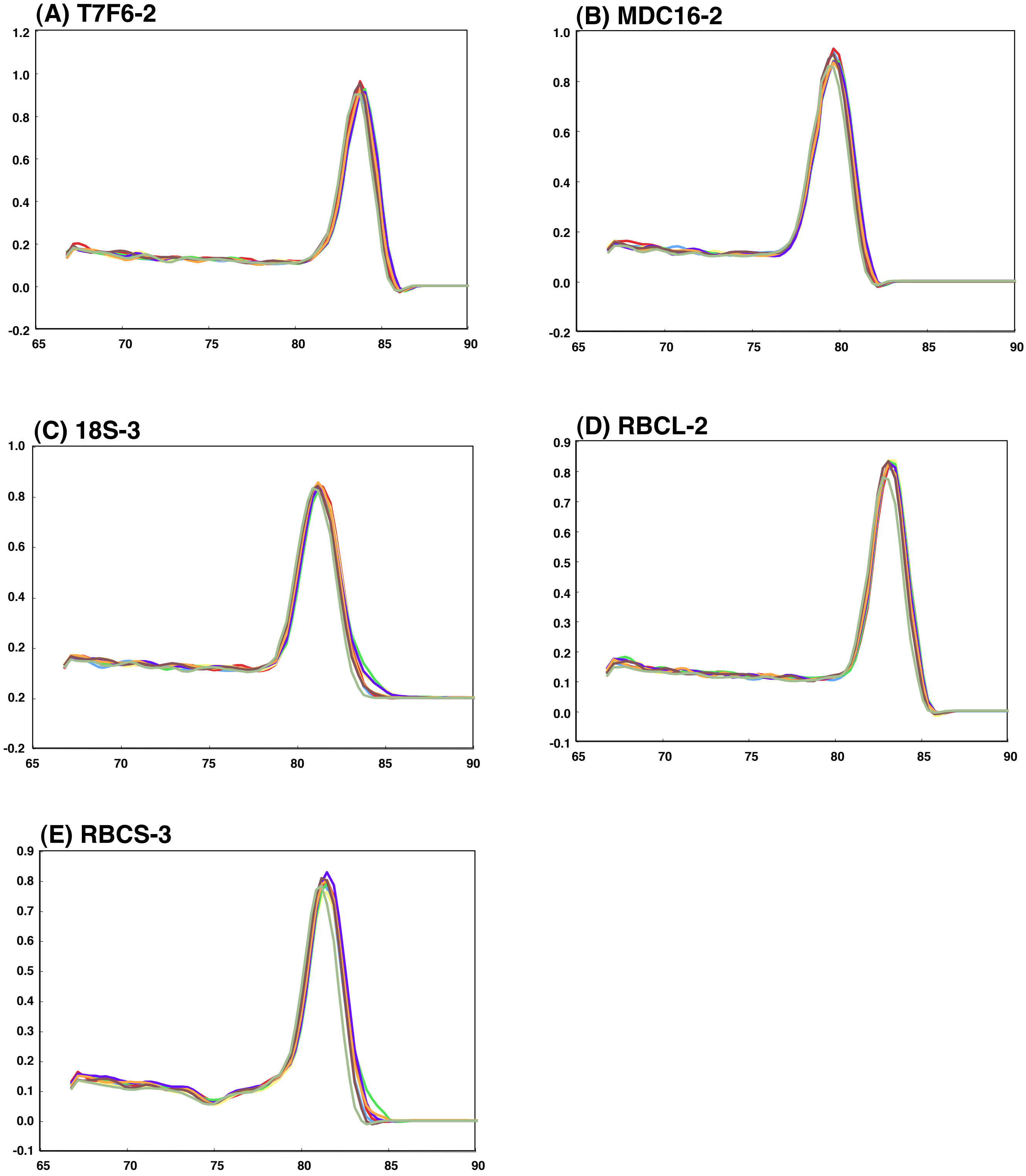

Additional File 3 Dissociation curves. Dissociation curves for the PCR products generated using the primer set of T7F6-F-2 and T7F6-R-2 (A), MDC16-F-2 and MDC16-R-2 (B), 18S-3-F and 18S-3-R (C), RBCL-2-F and RBCL-2-R (D), or RBCS-3-F and RBCS-3-R (E) (see additional file 1). The *y* axis shows the logarithm of fluorescence. These curves reflect normalized data.
