# Supplementary material for: Normalization using ploidy and genomic DNA copy number allows absolute quantification of transcripts, proteins and metabolites in cells
Source: Plant Methods. 2010 Dec 29;6:29. doi: 10.1186/1746-4811-6-29 (PMC3023742; doi:10.1186/1746-4811-6-29)
Supplement: Additional File 4 — Transcript number per cell in 3-week-old rosette leaves of wild-type Arabidopsis. Table shows the transcript number of RBC-L, RBC-S, 18S, ACT2, PDF2, SAND, GAPDH, UBC, EF-1α, PPR, YLS8 and UBC9 genes per cell. [file 1746-4811-6-29-S4.PDF]

Additional File 4
Transcript number per cell in 3-week-old rosette leaves of wild-type Arabidopsis

Gene	Transcripts / cell	*+s.d.*	*-s.d.*
RBC-L	7.5×10^3	1.8×10^3	1.5×10^3
RBC-S	9.9×10^3	2.5×10^3	2.0×10^3
18S	1.4×10^6	0.3×10^6	0.2×10^6
ACT2	1.2×10^2	0.3×10^2	0.2×10^2
PDF2	9.8	2.4	2.0
SAND	4.5	1.0	0.8
GAPDH	2.4×10^2	0.7×10^2	0.6×10^2
UBC	7.5	1.8	1.4
EF-1a	2.7×10^2	0.6×10^2	0.5×10^2
PPR	0.17	0.06	0.04
YLS8	29	6	5
UBC9	27	6	5
