# Supplementary material for: Normalization using ploidy and genomic DNA copy number allows absolute quantification of transcripts, proteins and metabolites in cells
Source: Plant Methods. 2010 Dec 29;6:29. doi: 10.1186/1746-4811-6-29 (PMC3023742; doi:10.1186/1746-4811-6-29)
Supplement: Additional File 5 — Standard curve indicating the Ct relative the dilution of protein extract sample from a wild-type plant as template for qPCR. One microliter of wild-type protein extract (diluted 1:10, 1:20, 1:40, 1:80, or 1:160) was used as template for qPCR. [file 1746-4811-6-29-S5.PDF]

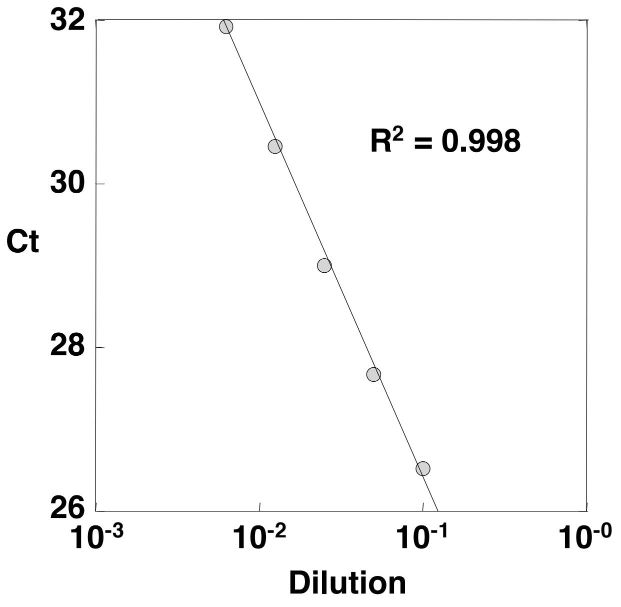

Additional File 5 Standard curve indicating the Ct relative the dilution of protein extract sample from a wild-type plant as template for qPCR. One microliter of wild-type protein extract (diluted 1:10, 1:20, 1:40, 1:80 or 1:160) was used as template for qPCR.
