# Supplementary material for: Normalization using ploidy and genomic DNA copy number allows absolute quantification of transcripts, proteins and metabolites in cells
Source: Plant Methods. 2010 Dec 29;6:29. doi: 10.1186/1746-4811-6-29 (PMC3023742; doi:10.1186/1746-4811-6-29)
Supplement: Additional File 6 — Calculation of genomic DNA copy number in protein extracts. This file shows mathematical formula with actual command sequences on R language to calculate genomic DNA copy number. [file 1746-4811-6-29-S6.PDF]

Additional File 6 Calcualtion of genomic DNA copy number in protein extracts. To estimate genomic DNA copy number, we assessed the effect of an added external plasmid DNA on PCR amplification of a specific segment of genomic DNA. This PCR amplification process can be formulated as follows:

$(g + p)(2E)^{Ct} = A$, where g is the genomic DNA copy number, p is the plasmid copy number, Ct is the PCR cycle number, E is the PCR amplification coefficient, and A is the number of amplified molecules. This equation was solved for Ct as follows: $Ct = \frac{logA - log(g + p)}{log2E}$

The nonlinear least-squares method was used to obtain the parameters g, E and A (R language; http://www.r-project.org).

A function "nls" for nonlinear least-squares method on R language (http://www.r-project.org) was used to estimate unknown parameters on the PCR amplification model written in the manuscript. Genome content ***g*** was estimated as one of the unknown parameters.

```
# data input (example for  Ws-3)
# x for plasmid  p
# y for Ct
x <- c(2.882e+9, 2.882e+8, 2.882e+7, 2.882e+6, 2.882e+5, 2.882e+4, 2.882e+3, 2.882e+2,
2.882e+9, 2.882e+8, 2.882e+7, 2.882e+6, 2.882e+5, 2.882e+4, 2.882e+3, 2.882e+2, 0e+0,
0e+0, 0e+0,  0e+0)
y <- c(11.06, 14.13, 17.81, 22.02, 25.73, 29.16, 30.82, 30.95, 11.09,
14.33, 18.27, 22.15, 25.99, 29.15, 30.72, 31.26, 31.33, 31.39, 31.24,
31.31)

# estimation of parameters a, b, c by nonlinear least-squares method
# a for  A
# b for beta
# c for genome contents  c
ans <- nls(y~ (log10 (a) - log10 (c + x)) / (log10(2*b)), start=c(a=15,b=1,c=5))

# output
summary(ans)
```
