# Supplementary material for: Normalization using ploidy and genomic DNA copy number allows absolute quantification of transcripts, proteins and metabolites in cells
Source: Plant Methods. 2010 Dec 29;6:29. doi: 10.1186/1746-4811-6-29 (PMC3023742; doi:10.1186/1746-4811-6-29)
Supplement: Additional File 7 — Scheme for preparing cDNA and genomic DNA. Scheme shows the methods of extraction of the nucleic acid (genomic DNA and total RNA), DNase I digestion, reverse transcription and RNase digestion, and the extraction buffer composition. [file 1746-4811-6-29-S7.PDF]

Additional File 7 Scheme for preparing of cDNA and genomic DNA

Extraction of the nucleic acid (genomic DNA and total RNA)

Rosette leaves

↓ Homogenize in liquid nitrogen

+ 5 vol. Extraction buffer

+ 5 vol. phenol saturated with 1 M MOPS-KOH (pH 7.0)

+ 5 vol. chloroform : isoamyl alcohol (24:1)

↓ Vortex vigorously 5 min

Centrifugation at 20,000 xg 5 min

Extraction with phenol : chloroform : isoamyl alcohol (25 : 24 : 1) x 2 times

+ 2.5 vol. 100% ethanol, −80°C 30 min

Centrifugation at 20,000 xg 10 min

70% ethanol, rinse

Dry up 10 min

↓ + 50 μl of nuclease-free water

Total nucleic acid

Extraction buffer
100 mM MOPS-KOH (pH 7.0)
10 mM EDTA
0.3 M NaCl
1.0% SDS

DNase I digest (TURBO DNA-*free*™ Kit, Ambion)

4 μl of total nucleic acid (A_{260} = 20.0)

5 μl of 10X TURBO DNase Buffer

1 μl of TURBO DNase

40 μl of Nuclease-free Water

↓ 37°C 30 min

+ 1 μl of TURBO DNase

↓ 37°C 30 min

+ 10 μl of DNase Inactivation Reagent

↓ Mix, 2 min at room temperature

Centrifugation at 10,000 xg 1.5 min

↓ Transfer the RNA to a fresh tube

Total RNA

RNase digest (Wako)

4 μl of total nucleic acid (A_{260} = 20.0)

5 μl of TE

1 μl of RNase (20 ng/μl)

↓ 37°C 60 min

+ 51 μl of TE

↓

6.5 μl of RNase-free genomic DNA

3.5 μl of Nuclease-free Water

↓

Genomic DNA

Reverse transcription (PrimeScript® RT reagent Kit, Takara)

2μl of 5x PrimeScript® Buffer

0.5 μl of PrimeScript® RT Enzyme Mix

0.5 μl of Oligo dT Primer (50 μM)

0.5 μl of Random 6 mers (100 μM)

6.5 μl of Total RNA containing no genomic DNA

↓ 37 °C 15min, 85°C 5 sec, 4°C Stock

cDNA
