# Supplementary material for: Normalization using ploidy and genomic DNA copy number allows absolute quantification of transcripts, proteins and metabolites in cells
Source: Plant Methods. 2010 Dec 29;6:29. doi: 10.1186/1746-4811-6-29 (PMC3023742; doi:10.1186/1746-4811-6-29)
Supplement: Additional File 8 — Agarose gel analysis of total nucleic acid, genomic DNA, and total RNA preparations. Total nucleic acid from wild-type plants was treated without (lane 1) or with RNase (lane 2) or DNase (lane 3) and subjected to 1.2% agarose gel electrophoresis followed by ethidium bromide staining. [file 1746-4811-6-29-S8.PDF]

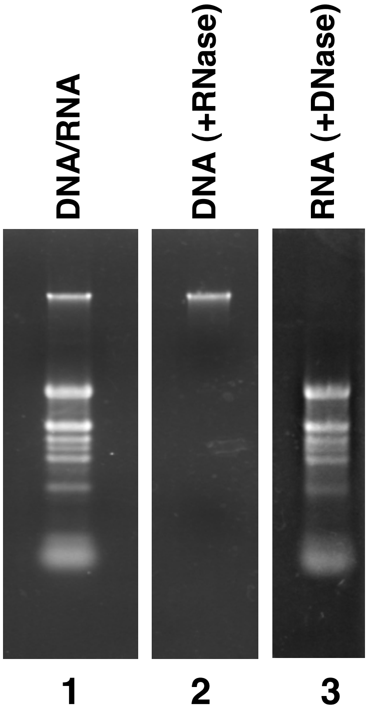

Additional File 8 Agarose gel analysis of total nucleic acid, genomic DNA, and total RNA preparations. Total nucleic acid from wild-type plants was treated without (lane 1) or with RNase (lane 2) or DNase (lane 3) and subjected to 1.2% agarose gel electrophoresis followed by ethidium bromide staining.
